# Self-Reported Diet Quality Differentiates Nutrient Intake, Blood Nutrient Status, Mood, and Cognition: Implications for Identifying Nutritional Neurocognitive Risk Factors in Middle Age

**DOI:** 10.3390/nu12102964

**Published:** 2020-09-28

**Authors:** Lauren M. Young, Sarah Gauci, Andrew Scholey, David J. White, Annie-Claude Lassemillante, Denny Meyer, Andrew Pipingas

**Affiliations:** 1 Centre for Human Psychopharmacology, Swinburne University, Melbourne, VIC 3122, Australia; laurenyoung@swin.edu.au (L.M.Y.); sarahgauci@swin.edu.au (S.G.); andrew@scholeylab.com (A.S.); dawhite@swin.edu.au (D.J.W.); 2 Department of Nursing and Allied Health, Faculty of Health, Arts and Design, Swinburne University, Melbourne, VIC 3122, Australia; alassemillante@swin.edu.au; 3 Department of Health Science and Biostatistics, Centre for Mental Health, Swinburne University, Melbourne, VIC 3122, Australia; dmeyer@swin.edu.au

**Keywords:** diet quality, nutritional risk, nutrient intake, nutrient status, cognition, mood, stress, diet screening, middle-aged adults

## Abstract

Evidence for diet quality representing a modifiable risk factor for age-related cognitive decline and mood disturbances has typically come from retrospective, cross-sectional analyses. Here a diet screening tool (DST) was used to categorize healthy middle-aged volunteers (*n* = 141, 40–65 years) into “optimal” or “sub-optimal” diet groups to investigate cross-sectional associations between diet quality, cognitive function, and mood. The DST distinguished levels of nutrient intake as assessed by Automated Self-Administered 24-h dietary recall and nutrient status, as assessed by blood biomarker measures. Compared with the “sub-optimal” group, the “optimal” diet group showed significantly higher intake of vitamin E (*p* = 0.007), magnesium (*p* = 0.001), zinc (*p* = 0.043) and fiber (*p* = 0.015), higher circulating levels of vitamin B6 (*p* = 0.030) and red blood cell folate (*p* = 0.026) and lower saturated fatty acids (*p* = 0.012). Regarding psychological outcomes, the “optimal” diet group had significantly better Stroop processing than those with a “sub-optimal” diet (*p* = 0.013). Regression analysis revealed that higher DST scores were associated with fewer mood disturbances (*p* = 0.002) and lower perceived stress (*p* = 0.031), although these differences were not significant when comparing “optimal” versus “sub-optimal” as discrete groups. This study demonstrates the potential of a 20-item diet screen to identify both nutritional and psychological status in an Australian setting.

## 1. Introduction

The global cost of dementia was estimated in 2015 to be US $818 billion [1]. Given the ageing population, particularly in Western countries, this financial burden is projected to rise, as the number of people with dementia is expected to increase from 47 million people in 2015 to 75 million by 2030 [1]. As well as the financial burden to the health care system, the impact of dementia is detrimental to the individuals themselves. Adjustment to a new identity, loss of independence and disconnect from previous social interactions also contributes to increased vulnerability for mood disturbances and disorders with ageing. The neurobiological underpinnings of depression in particular appear to share similar characteristics as the cognitive deficits in ageing [2,3,4]. Unfortunately, pharmacological agents have been unsuccessful in slowing brain ageing and have only been able to alleviate the symptoms of these disorders. Further, nearly all agents are associated with an extensive list of side effects. Thus, there is an urgent need to understand risk factors throughout the lifespan which could be modified to prevent the onset of disease before they occur and to mitigate the negative impact of ageing on the brain.

In addition to the issues underlying an ageing population, there has been a shift in the dietary landscape from ‘traditional’ diets primarily composed of plant-based foods and minimally-processed meats toward modern, ‘Western’ diets high in animal foods and ultra-processed foods. Modern ‘Western’ diets are high in calories but insufficient in essential micronutrients and low in phytonutrients [5], adversely affecting the systemic health of people of all body mass index (BMI) levels [6,7]. This dietary shift may also have consequences for brain health due to insufficiencies in the nutrients that support the maintenance of physiological processes required for optimal brain function, including; energy production, neurotransmitter regulation, inflammatory pathways and clearance of waste products [8,9,10]. Thus, insufficiencies in the bioavailability of micronutrients such as B vitamins may result in one or many of these processes being disrupted, leading to poorer brain health in the long term.

Diet is now being investigated beyond its benefits for physical health, and is increasingly recognized as a promising modifiable risk factor to protect against mood disturbances and age-associated cognitive decline [11,12,13,14,15,16,17,18]. Inter-individual differences in age-associated brain dysfunction provide opportunity to characterize the relationship between diet quality and psychological outcomes in middle age, prior to the presence of significant brain pathology. If diet quality is associated with sub-optimal cognitive and mood functioning in mid-life, it would strengthen the evidence for a nutrient-poor “Western” diet as a precursor to brain diseases in later life.

The majority of investigations related to diet quality have been limited to younger (<40 years) and older adults (>65 years), or clinical groups [19,20,21,22,23]. The study of younger adults may be hindered by psychological functioning already at an optimal level and therefore less amendable to change. Further, the effect of diet on brain health appears to be cumulative across the lifespan and thus may not be detectable at this early life stage. At the other end of the spectrum, older adulthood is characterized by a greater amount of neural insults on the brain. Diet alone is unlikely to reverse this damage, nor can it address problematic co-morbidities associated with senescence [24]. There is little research examining the effect of diet quality on cognitive and mood outcomes in non-clinical, middle-aged populations. Middle age presents an at-risk life-stage where mood and cognitive deficits associated with poor diet may begin to be detected [24]. Studying mid-life may enhance our ability to detect differences in cognitive and mood outcomes that are attributable to diet quality and may be an indicator of unhealthy brain ageing in later life.

This field of research has been further hindered by measuring diet quality post-hoc. This can lead to cohorts who have a narrow range in terms of adherence to a diet pattern, limiting the analysis available to researchers and consequently the ability to discriminate differences attributable to diet quality. Researchers have reported an overall high diet quality of study participants, suggesting a degree of self-selection (and associated bias) in volunteers who have an underlying interest in nutrition [25,26,27]. Given that the overall diet quality of people in Western countries is poor [5], there is a lack of research which attempts to recruit individuals with poorer diets and who are representative of the population. In order to ensure a diverse range of dietary patterns across trial participants, one option is for researchers to pre-screen participants based on their diet prior to inclusion in the study.

The Diet Screening Tool (DST) was developed as a broad screening tool to determine nutritional risk and to identify individuals for primary and secondary prevention of disease [28]. This approach offers clinical utility as health care practitioners can easily administer the 20-item screening tool and it does not require the cost and labor associated with more comprehensive methods of dietary assessment (24-h recalls and food frequency questionnaires). The DST has previously been validated in middle-aged adults, demonstrating its ability to discriminate across a range of nutrient intake and biomarkers [29]. However, studies using this tool have focused on general health outcomes [29,30]. It is unknown whether diet quality as determined by the DST is also associated with psychological outcomes of cognition and mood. If such a relationship does exist, the DST could be utilized to not only identify those at-risk of nutritional deficiency but those who may also be at-risk for poorer psychological outcomes in later life. Finally, studies using the DST have been exclusively conducted in the United States. Applicability of this tool for use in Western countries outside of the United States are yet to be tested.

The present study aimed to investigate the cross-sectional relationship between diet quality, nutritional intake, biochemical markers, cognitive function, and mood, in a healthy, middle-aged sample (40–65 years). Through the recruitment and screening of participants with both “optimal” (≥60 on DST) and “sub-optimal” (≤59 on DST) diets, this study aimed to recruit a cohort of participants with a diverse range of dietary intakes and to evaluate whether meaningful differences in psychological outcomes could be ascertained from this relatively simple measure of diet quality. It was hypothesized that in comparison to individuals with “sub-optimal” diets, individuals with “optimal” diets would have better cognitive performance and mood. As this is the first application of the DST nutritional risk categories to detailed measures of psychological outcomes, examination of the DST as a continuous variable was also examined to determine if any additional information could be derived from examining the full range of DST scores. A further aim of the study was to examine the validity of the Australian version of the DST by comparing this assessment with both 24-h dietary recall data and levels of circulating nutrients.

## 2. Materials and Methods

### 2.1. Study Population

The data for this study was sourced from the Memory and Attention Supplement Trial (MAST) conducted by researchers at the Centre for Human Psychopharmacology at Swinburne University of Technology. The trial was registered with the Australian and New Zealand Clinical Trials Registry, and ClinicalTrials.gov (NCT03482063). All participants completed written informed consent before they participated in the study and the research protocol was approved by the Swinburne University Human Research Ethics Committee (2017/269). This paper is restricted to the baseline data but briefly, the randomized control trial (RCT) aimed to investigate the efficacy of a 12-week intervention of a B vitamin and herbal supplement on memory, attention and mood and whether diet quality moderated any effect of the intervention. In order to ensure a broad range of diet quality, the study recruited 50% of participants with a “sub-optimal” diet and 50% with an “optimal” diet, as determined by the DST [28]. This paper presents the baseline data to address the question of whether a relationship exists between baseline diet quality and psychological outcomes of cognition and mood. Results of the RCT will be presented elsewhere.

**Figure 1:**
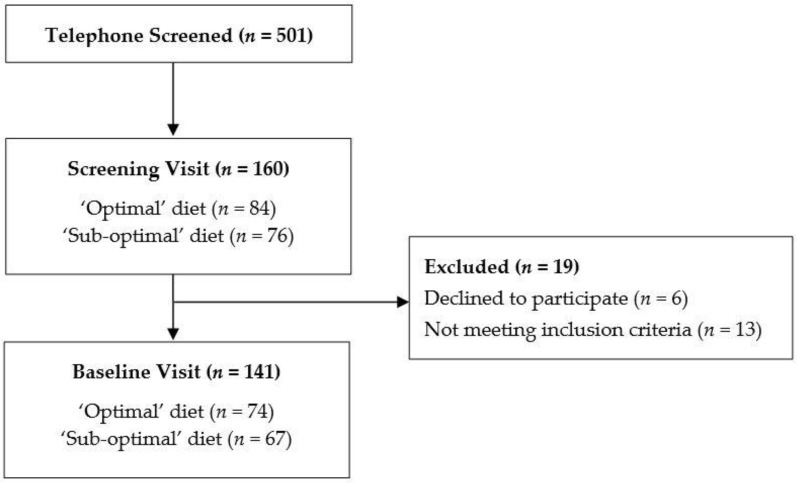
Flowchart of participants included in the study.

Participants were recruited from the community between May 2018 and September 2019. Online advertising and posters around the university were used to recruit participants aged between 40 and 65 who were in good general health. To be eligible to participate in the study, participants must have been free from neurological conditions, cognitive impairment, mood or psychiatric disorders, any health conditions that may affect the absorption of food and given the nature of cognitive tasks, must not be color blind. Participants were excluded if they were taking medication, herbal extracts, vitamin supplements or illicit drugs that might reasonably be expected to interfere with cognition. Female participants could not be pregnant or lactating. Of the 501 participants telephone-screened, 160 completed an initial visit in the laboratory where eligibility was confirmed. One hundred and forty-one participants completed a baseline assessment, and 116 completed the follow up assessments. Here we present a cross-sectional analysis of participants with complete baseline data (*n* = 141), collected prior to any intervention (Figure 1).

**Figure 2:**
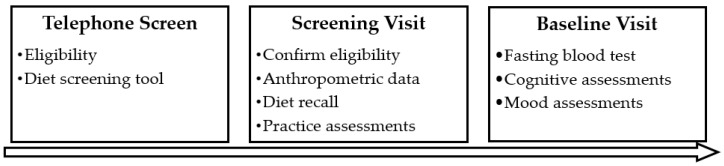
Schedule of Assessments.

Following successful telephone screening, participants were invited to attend a screening and study enrolment visit at Swinburne University in Hawthorn, Melbourne. They gave written consent and were further screened for eligibility including for cognitive decline using the Mini Mental State Examination (scores < 24 excluded), mood disturbances using the Beck Depression Inventory (scores > 20 excluded) and blood pressure using the SphygmoCor^®^ device (Model XCEL, AtCor Medical, Sydney, Australia; systolic < 160 mmHg, diastolic < 100 mmHg). Anthropometric data were collected, including the calculation of BMI from weight in kilograms divided by height in m^2^. Participants completed a practice version of the cognitive and mood assessments and their first 24-h diet assessment with the researcher. Participants were instructed to complete a second 24-h diet assessment prior to their next (baseline) visit, which was scheduled within two weeks of their first visit. For their baseline visit, participants were instructed to fast from 10pm the night before, and to abstain from alcohol and caffeine 12 h before. At arrival at the laboratory (between 8–10:30 a.m.), while fasted, participants had a blood sample taken and were then provided with a standardized breakfast and short break. Participants then completed their baseline cognitive and mood assessments which are detailed below. An outline of assessments is displayed in Figure 2.

### 2.2. Cognitive Assessments

Participants completed the Swinburne University Computerized Cognitive Assessment Battery (SUCCAB) which is designed to capture cognitive functions that are vulnerable to ageing [31] and has been shown to be sensitive to dietary supplementation [32,33]. Participants completed a short practice before each task. The battery consists of eight tasks which are described below, and responses were recorded with a button-box.

For each cognitive task, participants were asked to respond as quickly and as accurately as possible. Accuracy (% of correctly performed trials) and average response time for correctly performed trials (in milliseconds) were used to calculate performance scores (accuracy/response time) which accounts for the speed/accuracy trade off seen with age [34,35]. A higher performance score was indicative of better performance. In order to reduce the number of measures and risk of Type I error, performance scores on each task were mapped to form four cognitive domains; Reaction & Decision Speed, Visual Processing, Spatial Working Memory and Stroop Processing. Computation of these scores have been described elsewhere [36]. Briefly, “Reaction & Decision Speed” was derived from the average performance for the Simple Reaction Time and Choice Reaction Time tasks; “Visual Processing” was derived from the average performance for the Immediate Recognition, Delayed Recognition and Contextual Memory tasks and “Spatial Working Memory” was derived from the average performance for the Spatial Working Memory task. The final cognitive domain, “Stroop Processing” was derived from the difference between Incongruent and Congruent Stroop performance to provide a measure of inhibitory control. Performance on each task was converted to Z scores prior to computing domain scores to ensure each task contributed equal weighting to their respective cognitive domain. For each cognitive domain, higher scores indicated better performance.

#### 2.2.1. Simple Reaction Time

Participants responded with a right button press to the appearance of a single white square at the center of the screen. Thirty targets were presented with a randomized inter-stimulus interval (ISI) to avoid anticipation effects.

#### 2.2.2. Choice Reaction Time

Participants completed 20 trials which required them to respond with a left (blue) or right (red) button press to the appearance of a blue triangle or red square respectively. Presentation order and ISI were randomized to avoid anticipation effects.

#### 2.2.3. Immediate/Delayed Recognition

Participants were asked to study a series of 40 abstract images presented serially in the center of the screen for three seconds each with no ISI. On completion, another series of images were presented, half of which were from the studied series and half that were new (Immediate condition). Participants indicate with a right (yes) or left (no) button press whether or not they recognized the image from the studied series. This task was repeated at the end of the testing session with the remaining 20 images from the studied series and another 20 new images (Delayed condition).

#### 2.2.4. Stroop Color-Word

The test consisted of congruent and incongruent trials blocks. Stimulus words were randomly presented (RED, BLUE, GREEN, YELLOW), printed in colored font either congruent or incongruent with the written word. Participants are asked to respond by pressing one of four buttons corresponding to the color of the font while ignoring the written word. The tasks are participant-paced, meaning that as soon as a participant responds to a word, they are presented with the next word.

#### 2.2.5. Spatial Working Memory

In each trial participants were presented with a 4 × 4 white grid on a black background, with six grid positions containing white squares. Participants were given three seconds to remember where the white squares were located. The grid became blank and a series of four white squares were sequentially superimposed on the grid in one of the 16 positions. Participants responded with a yes/no response to indicate whether each square matched a position that was originally filled. In total, participants complete 14 trials, each of which are separated by a blank screen displayed for two seconds. Each trial was set such that two out of the four locations in the response series corresponded to the original grid locations, and two did not. The task required participants to hold spatial information in working memory.

#### 2.2.6. Contextual Memory

A series of 20 everyday images were presented at the top/bottom/left/right of the screen for three seconds each with no ISI. On completion of the series the same images were displayed again in randomized order in the center of the screen. Participants respond with a top/bottom/left/right button press to indicate the original location of each image. The task required participants to recall the spatial context of the original presentation and was used as a measure of episodic memory.

### 2.3. Mood Assessments

Mood was assessed with the Profile of Mood States (POMS) and Perceived Stress Scale (PSS). The POMS required participants to indicate the degree to which they have identified with 65 mood-related adjectives over the past week. Each item was on a five-point scale from “not at all” to “extremely”. Items are summed into six factors; Tension-Anxiety, Confusion-Bewilderment, Anger-Hostility, Depression-Dejection, Fatigue-Inertia and Vigor-Activity. A Total Mood Disturbance score was computed as the sum of the first 5 factors minus Vigor-Activity, with higher scores indicative of greater mood disturbance. 

The PSS measured the extent to which respondents have perceived events in their life as stressful over the last month. Each of the 10 items were scored on a five-point scale ranging from “never” to “very often”. Four of these items are positively stated items, and therefore they are reverse scored. Higher scores on the PSS were associated with higher levels of perceived stress.

### 2.4. Diet Assessment

In order to determine diet quality, prior to enrolment, participants were asked questions from the Australian-adapted version of the DST [37]. Originally developed by Bailey et al. [28], the tool has been used to identify nutritional risk in community-dwelling older adults as a predictor of vitamin levels. The 20-item questionnaire had a maximum score of 104, with higher scores indicating better diet quality, and less nutritional risk. Eighteen items related to the frequency of consumption of particular foods (e.g. “how often do you consume legumes, such as lentils or chickpeas?”), and two items related to the number of servings consumed (e.g. “how many different vegetable servings do you usually have at your main meal of the day?”). While the original DST had three nutritional risk categories; ”at-risk”, “possible-risk” and “not-at-risk” [28], the present study utilized a binary grouping of diet quality to resemble the previous validation of the tool in middle-aged Appalachian adults [29]. In the present study, participants scoring ≤ 59 were classified as having a “sub-optimal” diet reflecting less optimal food intake; participants scoring ≥ 60 were classified as an “optimal” diet reflecting a more nutrient-dense diet [38]. These cut-off scores have previously been used to identify nutritional risk, with those scoring ≤ 59 having lower intake of micronutrients, fiber, fruit, vegetables and lower nutritional status including Vitamin B12 and folate [28]. The study utilized a novel recruitment strategy which aimed to a-priori recruit 50% of participants for each dietary group and then compare them across a range of psychological outcomes.

Once enrolled in the study, participants completed a series of Automated Self-Administered 24-h Dietary Assessments (ASA24). ASA24 required participants to record the foods, drinks and dietary supplements they consumed over 24 h, even if they did not reflect their usual diet. Nutrient intakes were calculated from the nutrient composition data from Australian Food, Supplement and Nutrient Database (AUSNUT) 2011–2013. Data from two ASA24 recalls (one completed at in the lab at the screening visit, one completed at home prior to the baseline visit) were used to compare nutrient intakes across DST groups (“optimal” vs. “sub-optimal”).

### 2.5. Biochemical Markers

Blood-borne markers of nutrients and total lipid fatty acids were used to compare circulating levels across DST groups (“optimal” vs. “sub-optimal”). Participants fasted from 10pm the night before their baseline visit. Fasting venous blood samples were collected by a qualified venipuncture technician or research nurse at the Centre for Human Psychopharmacology. A serum separator tube (8.5 mL) containing clot activator (silicone and micronized silica) was allowed to clot at room temperature for 30 min before being centrifuged for 10 min at 4000 rpm and then analyzed for homocysteine and Vitamin B12. A lithium heparin tube (6 mL) was immediately wrapped in aluminum foil to prevent degradation of the sample by light and analyzed for Vitamin B6. An EDTA tube (4 mL) was stored at room temperature and then analyzed for red blood cell (RBC) folate. All samples were pre-processed on site before being sent by courier to a commercial pathology laboratory for analysis. Due to processing errors, a reduced sample size was available for RBC folate. 

A second 10 mL sample was collected in an EDTA tube for total lipid fatty acid analysis. The sample was centrifuged for 5 min at 4 °C and 4000 rpm. Plasma and buffy coat were removed, and the packed RBC were washed with 0.9% saline by few gentle inversions. The tube was then centrifuged at 4 °C and 4000 rpm for 5 min, and the supernatant was discarded. This process was repeated twice. The packed RBC samples (approximately 1 mL) were stored in −80 °C freezer before being couriered and processed by South Australian Health and Medical Research Institute (SAHMRI) Fatty Acid Laboratory (Adelaide, Australia).

### 2.6. Statistical Analysis

All analyses were performed using IBM SPSS Statistics for Windows version 26 (IBM Corp., Armonk, NY, USA). Demographic information was reported using means and standard deviations, or medians and interquartile range (IQR) for non-normally distributed variables, for all participants and by diet group. Variables were screened for normality prior to tests for group differences. Positively skewed variables (nutrient intake and Total Mood Disturbance) were transformed using the square root or logarithmic function. Negatively skewed variables (Stroop Processing) was transformed using the square function. To screen for outliers, Z scores were calculated for each variable and displayed in histogram form. Z scores greater than 3.29 and/or greatly disconnected from the data spread were designated as outliers and excluded from analysis. Energy over- and under-reporting was checked, and one participant was excluded from nutrient intake analysis for implausible daily energy intake (>5000 kcal/day). Accuracy operating below chance for cognitive tasks were considered indicative of not understanding the task requirements and were excluded from analysis for that task (*n* = 2).

The purpose of this analysis was to compare “optimal” and “sub-optimal” diet groups across demographic variables, nutrient intake, blood biomarkers and psychological outcomes. Difference in demographics across diet groups were tested using analysis of variance for continuous variables and chi-square tests for categorical variables. Any demographic differences detected between diet groups were carried through as control variables for all subsequent analysis. Mean dietary intake of nutrients were calculated as the average across the two 24-h diet recalls. Nutrient intake was adjusted for overall energy intake by dividing overall nutrient intake by total energy (kcal). This attempted to account for differences in energy requirements across sex, body size and metabolic efficiency. Energy adjusted nutrient intake, biomarkers of nutritional status, cognitive and mood outcomes were compared across diet groups using analysis of covariance (ANCOVA). Statistical significance was set at *p* < 0.05.

#### 2.6.1. Covariates

Analysis of the relationship between the demographic variables and the diet groups revealed that both age at enrolment and BMI differed significantly between groups and they were subsequently controlled for in all analyses. ANCOVA models concerning cognition and mood were also controlled for gender and education due to their influence on psychological outcomes [39]. Education was based on self-reported years of formal schooling (or the full time equivalent). As this group of middle-aged adults captured women across varying stages of hormonal status, these differences were explored post-hoc.

#### 2.6.2. Exploratory Analysis

While recruitment of participants was stratified into discrete categories of “optimal” and “sub-optimal” diet groups, the cut-off point used to define these groups has not been validated in an Australian population. Further, the utility of the DST and these cut-off points when applied to psychological outcomes is yet to be tested. Therefore, separate regression analyses were conducted with cognitive and mood outcomes entered as the dependent variable and DST as the independent variable. This analysis allows exploration of whether additional information could be derived from examining the full range of DST scores in relevance to psychological outcomes rather than its previous use as a categorical variable with pre-defined cut-offs. Residual plots obtained from these analyses were inspected to ensure the assumptions of normality and homoscedasticity were supported. Multivariate outliers were screened using Mahalanobis Distances and none were identified.

Finally, there is increased consensus that the relationship between diet and the brain is complex and engages multiplicative mechanisms [40]. However, studies of diet quality often utilize parametric modelling techniques which assume a linear relationship between diet quality and psychological outcomes. As the true nature of the relationship between diet quality and psychological outcomes is still unknown, the present study sought to further explore these relationships. In the event of a significant relationship detected between the DST and a psychological outcome, locally weighted smoothing scatterplots (LOESS) were generated to explore the data in an attempt to reveal the true nature of these relationships which may be overlooked with traditional, parametric modelling procedures [41].

## 3. Results

### 3.1. Sample Characteristics

Sample descriptives are presented in Table 1. Individuals with an “optimal” diet were older and had a lower BMI than those with a “sub-optimal” diet. Females with an “optimal” diet were more likely to be post-menopausal, while women with a “sub-optimal” diet were more likely to be menstruating (note the significant group difference in age). There were no other significant differences across demographic variables.

### 3.2. Nutrient Intake

Energy-adjusted nutrient intake derived from the ASA-24 across all participants and by diet quality group are presented in Table 2. All following analyses were controlled for age and BMI due to baseline differences across diet quality groups. Participants with an “optimal” diet had a lower intake of carbohydrates (*p* = 0.027) and higher intake of fiber (*p* = 0.015) than individuals with “sub-optimal” diets. The “optimal” diet group also had significantly higher intake of Vitamin E (*p* = 0.007), Magnesium (*p* = 0.001) and Zinc (*p* = 0.043) than those with “sub-optimal” diets. In terms of fatty acid intake, those with an “optimal” diet had higher intake of polyunsaturated fatty acids (PUFA, *p* = 0.001) and monounsaturated fatty acids (MUFA, *p* = 0.017) than those with a “sub-optimal” diet. Examination of PUFA profile found that those with “optimal” diets had higher intake of omega-6 (*p* = 0.001) and higher omega 6:3 ratio (*p* = 0.044).

### 3.3. Biochemical Markers

As presented in Table 3, individuals with an “optimal” diet had higher circulating levels of Vitamin B6 (*p* = 0.030) and RBC folate (*p* = 0.026), and lower saturated fatty acids (SFA, *p* = 0.012) than those with a “sub-optimal” diet. Consistent with nutrient intake, those with an “optimal” diet had a higher omega-6 to omega-3 ratio than those with a “sub-optimal” diet, although this failed to reach significance (*p* = 0.052).

### 3.4. Psychological Outcomes

Table 4 presents a comparison of differences for cognitive domains and mood by diet quality group. All analyses were adjusted for age, gender, education and BMI. Individuals with an “optimal” diet had significantly better Stroop Processing than those with a “sub-optimal” diet (*p* = 0.013). There were no other significant differences for psychological outcomes across diet quality groups. Post-hoc analysis considering hormonal status in females found no significant difference.

**Figure 3:**
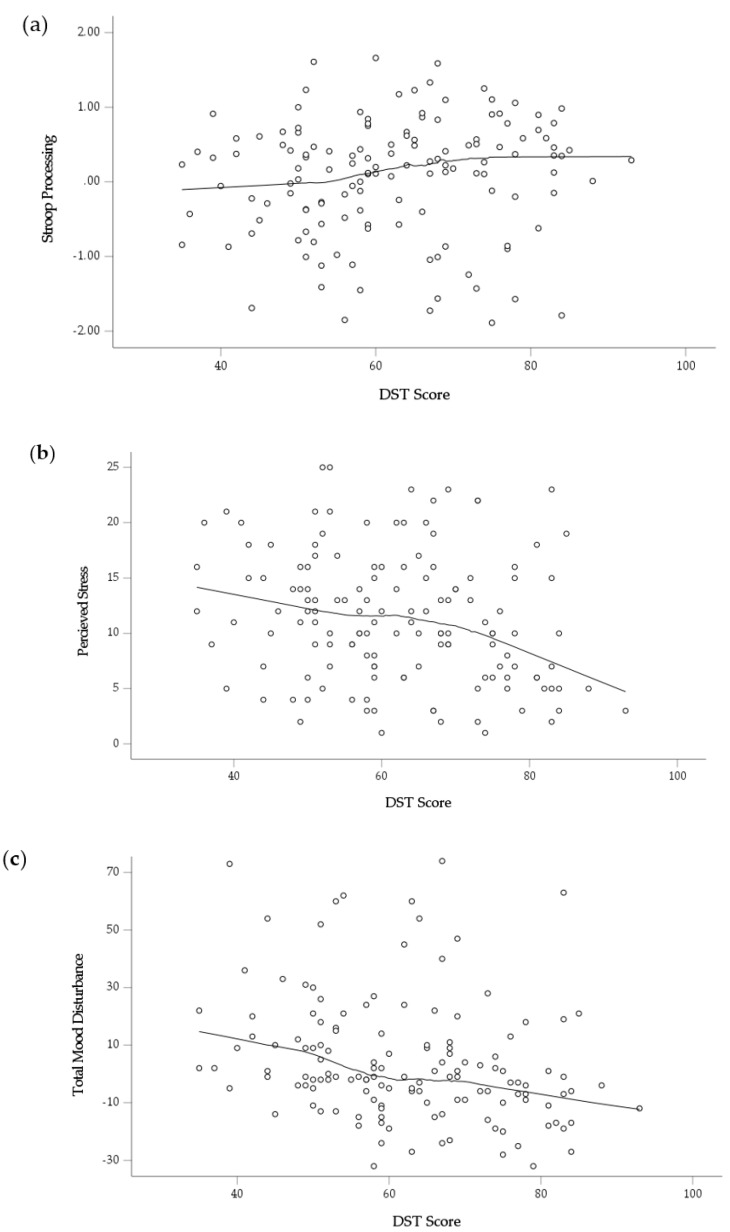
Locally weighted smoothing scatterplots demonstrating how diet quality (DST score) is related to (**a**) Stroop Processing (Z score, difference between Incongruent and Congruent performance); (**b**) Perceived Stress; (**c**) Total Mood Disturbance.

Finally, in contrast to the ANCOVA models which compared diet quality across discrete “optimal” and “sub-optimal” groups, analysis of the DST as a continuous variable revealed that diet quality was significantly associated with mood outcomes, but not cognition. After controlling for covariates, higher DST scores were associated with lower perceived stress (*p* = 0.031) and Total Mood Disturbance (*p* = 0.002) (Table 5). However, Stroop processing was no longer significant when diet quality was modelled as a continuous outcome (*p* = 0.166). This contrast indicates that diet quality may be differentially related to cognitive outcomes in comparison to mood outcomes. To explore these relationships further, LOESS were generated post-hoc to reveal the more complex relationships of diet with cognition and mood [41] (Figure 3). As shown in Figure 3a, Stroop processing appears to show a slight change in gradient at a score of approximately 55 on the DST before plateauing once the DST reaches 75. The rate of decline in perceived stress declines more sharply after a DST of 70 (Figure 3b) while the relationship between Total Mood Disturbance and DST shows a more consistent decline throughout the range of diet screening scores (Figure 3c).

## 4. Discussion

The present study was the first to extend the application of a diet quality screening tool beyond predicting nutritional status to determine how diet quality is associated with psychological outcomes. Detailed assessments of nutrient intake and biochemical markers revealed that the Australian version of the DST successfully distinguished middle-aged adults across a variety of nutrients. The targeted screening process employed in this study was able to capture a broad range of participants in terms of their diet quality and circulating level of nutrients. Specifically, assessment of biochemical markers found that those with “optimal” diets had higher circulating levels of Vitamin B6 and RBC folate and lower SFA than those with ‘sub-optimal’ diets. When considering psychological outcomes, the nature of the relationship between diet quality and the Stroop processing cognitive domain differed when compared to the relationship between diet quality and mood outcomes. While those with an “optimal” diet had significantly better Stroop processing than those with a “sub-optimal” diet, this effect did not hold when diet quality was modelled as a continuous variable. Conversely, higher DST scores were associated with both lower mood disturbance and perceived stress, but such differences were not significant when comparing “optimal” versus “sub-optimal” as discrete groups in ANCOVA models. Exploration of the data through the use of LOESS plots revealed that the relationship between the DST and various psychological outcomes (Stroop Processing, Perceived Stress, Total Mood Disturbance) varied, introducing the possibility that the relationship between diet and different psychological outcomes may not follow a consistent, linear trend.

This study had a number of novel aspects. By utilizing an Australian adapted version of the original DST, it was the first to measure nutrient intake and biochemical markers with relevance to dietary risk category outside of an American setting. The cut-off points of “optimal” and “sub-optimal” appeared sufficient in differentiating individuals who were characteristically different in terms of their nutrient intake and status. Similar to previous studies, the DST was able to discriminate across intakes of vitamin E, magnesium, zinc, PUFA and MUFA, with individuals with an “optimal” diet having greater intake of these nutrients and healthy fats [28,29]. Contrary to expectations, the breakdown of fatty acid intake revealed that those in the “optimal” diet group reporting significantly higher intake of omega-6 and a higher omega-6 to omega-3 ratio than those with a “sub-optimal” diet. Both of these measures are characteristic of a high adherence to a “Western” style diet. However, this did not translate into significant differences in circulating blood levels of PUFA as the small difference in mean was not likely clinically significant. The present paper extended on previous work to measure B vitamin and essential fatty acids. As expected with increased diet quality, circulating levels of Vitamin B6 and RBC folate were higher and SFA was lower in those with an “optimal” diet.

The use of a DST in lieu of blood tests holds promise as an approach in preventative medicine to identify those at nutritional risk and for future disease. The instrument takes less than 10 min to administer and score. If further validated in future studies, it could be applied to identify those at-risk, without the need for biochemical testing which is relatively costly, resource-intensive and invasive. The evidence from the present study demonstrates that the DST may also be sensitive to detect differences across B vitamin markers. Further research in larger sample sizes are needed to confirm this validity across a broader range of vitamins and minerals (vitamin D, calcium, vitamin E). In addition, the threshold for those “optimal” and “sub-optimal” are yet to be established in an Australian population. Sensitivity analysis should be conducted to determine if the cut-off point used is appropriate for use in this population.

Unlike objective biochemical markers, the DST score has the issue of social desirability bias. The tendency to over-report on foods that individuals perceive to be more favorably viewed can artificially inflate diet quality and skew the results [42]. This study was unique in its design in that it required a-priori screening for diet quality such that 50% of the sample were classified as having a “sub-optimal” diet. While this approach ensured a diverse range of diet quality, one cannot rule out potential bias within the included sample. Furthermore, the sensitivity by which the DST determines “nutritional risk” may vary across different nutrients. This issue is highlighted when considering the results for vitamin B12 where subgroup differences ran counter to the rest of the B group vitamins. Those who adhere more highly to a ‘Western’ style diet may be classified as “sub-optimal” according to the DST, but due to a high intake of animal-based foods will have a high intake of B12. In comparison, vegans or vegetarians are likely to be classified as “optimal” due to a high intake of plant-based foods but have a greater likelihood of B12 deficiency due to low levels of B12-rich foods in these dietary patterns. Indeed, this trend was observed in the present study with individuals with an “optimal” diet having lower levels of B12 (*p* = 0.153), while previous studies examining B12 intake across diet quality groups has been mixed [28,29]. Therefore, despite the promise of DST as an indicator of nutritional risk, the example of B12 asserts that biochemical testing cannot be replaced as the most objective measure of nutrient status.

Interestingly, diet quality improved with age in this middle-aged group. While increasing rates of protein-energy malnutrition has been related to the ageing process [43,44], this has been attributed to reduced oral intake, socio-economic challenges, co-morbidities and medication side-effects–all factors which are not applicable to our study participants who were in otherwise good health. In this middle-aged sample, there are a variety of factors that could lead to this positive association with diet quality. One suggestion is that individuals closer to the upper age bracket (65 years) are more likely to be visiting primary care settings (e.g., General Practitioners) than their younger counterparts (aged 40 years) and this may have led to them being more conscious of their health, and in turn, adhere to a more nutrient-rich diet. Due to this difference and broad range, age was controlled for in all analyses. Even after controlling for age, the present study found that the DST distinguished differences in nutrient intake and circulating nutrient status in these middle-aged adults.

Another novel aspect of this study was that it was the first application of the DST to detailed measures of psychological outcomes. Cognition and mood were differentially related to diet quality in this middle-aged group. While there was a significant difference between the “optimal” and “sub-optimal” diet groups for Stroop processing, diet groups did not differ for any other psychological outcomes. In contrast, when diet quality was modelled as a continuous variable, it was no longer associated with Stroop processing, however there was strong relationships with mood disturbances and stress. This differential relationship across cognitive and mood outcomes may be explained by the short-term and long-term effects of diet quality on the brain. Middle age represents an at-risk life stage when individuals may be vulnerable to unhealthy ageing. It has been proposed that the influence of diet on cognition is cumulative across the lifespan and perhaps assessment of individuals at this stage is too early to detect any detect any marginal differences attributable to diet quality. Further research in older adults may provide greater diversity in regard to the effect of diet quality on cognition in later life.

An interesting point to note is that the Stroop processing domain requires inhibitory control, specifically to inhibit the preponderance to process meaning over perceptual aspects of stimuli. There is evidence that inhibitory responses can predict unhealthy food choices [45,46] and therefore we may hypothesize that the relationship detected in the current study is a result of a bidirectional relationship. In other words, those with poor inhibitory control make unhealthy food choices leading to poorer diet quality. The relationship between diet quality and mood is also vulnerable to bi-directionality and this has been hypothesized as a contributor to why we typically see such strong relationships with mood in the absence of cognitive effects.

If we consider the LOESS graphs a model for the relationship that diet quality has with different psychological outcomes, the diversity of these relationships highlight a significant issue for the fields of cognitive neuroscience and nutritional psychiatry. Diet has a complex relationship with the brain that may not be consistent across different psychological outcomes. Further, in a randomized trial for diet improvement or supplementation, baseline diet quality may interact with the effect of an intervention [47]. The thorough analytical approach applied in the present study which considered diet quality as both a categorical and continuous variable revealed the complex relationships between diet and psychological outcomes that traditional statistical modelling procedures may overlook [41]. Future studies must exercise caution in the analysis and interpretation of diet-brain relationships, particularly when studying cognition and mood simultaneously.

While the nature of a cross-sectional study limits our ability to draw causality, there is a growing body of evidence supporting the intake of nutrient rich foods characteristic of a high-quality diet for optimizing brain function. The diet quality score used in the present study was modelled on the basis of nutritional risk for particular nutrients and was then modified to be appropriate to Australian foods. The food groups included in the 20-item questionnaire all have evidence either supporting or are detrimental to mental and cognitive health. In order to score highly on this diet tool, participants needed to consume a high intake of fruit, vegetables, legumes, olive oil and nuts which are known for their anti-inflammatory properties. Inflammation and oxidative stress are known pathways which cause progressive cognitive decline and hinder the biological pathways that regulate stress and emotional regulation [48,49]. This diet also supports a reduction in inflammatory foods (high intake of processed and red meats). It is unsurprising then that adherence to a diet supporting anti-inflammatory foods was associated with better cognitive and mood outcomes.

The present study is limited by a number of factors. First, despite the strong association between diet quality and mood outcomes, this study cannot disregard potential reverse causality. That is, does lower mood and poor inhibitory control result in decreased diet quality? A previous study in older adults found that life satisfaction and self-efficacy predicted DST risk category [30]. Several studies have attempted to determine the directionality of this relationship and have found mixed results. One study found a counterintuitive relationship, whereby past depression was associated with lower consumption of unhealthy foods in the future. In other words, poor mood in the past was associated with diet improvement in the future therefore it was ruled unlikely that poor mood caused poor diet in their sample [50]. A prospective study found contrasting results, reporting that chronic depressive symptoms in the past was associated with lower adherence to healthy diet patterns compared to individuals who had no depressive symptoms [20]. A recent meta-analysis found similar relationships for longitudinal and cross-sectional studies examining the relationship with diet and risk of depression [51], adding evidence to the argument of diet’s causal relationship with mood. It is still unclear the true nature of the diet-mood relationship, however it would be unwise to overlook the potential bi-directionality of these relationships.

Secondly, the DST provides an umbrella picture of diet quality and it does not capture every aspect of diet. More detailed approaches of studying various diet patterns including the Mediterranean diet may be more sensitive to capturing differences in cognitive performance. Additionally, we have only measured a select few biochemical markers that were relevant to the main RCT. There are many other nutrients (vitamin D, vitamin E) and non-nutrients including fiber which are important for brain health. Future research may choose to study the relationships between individual nutrients and psychological outcomes, however this was beyond the scope of the present paper.

Thirdly, it must be noted that the threshold for categorizing “optimal” versus “sub-optimal” has not be validated in the Australian version of DST. The differences in nutrient intake across the discrete diet groups provide compelling evidence that the tool has validity, however future studies should consider these specific cut-off points. We attempted to overcome this by conducting exploratory analysis of diet quality as a continuous variable which was able to reveal contrasting relationships with psychological outcomes.

Fourthly, as this study was largely exploratory, no Bonferroni correction was made. Due to the large number of comparisons across nutrient intake, biochemical markers and psychological outcomes, this study is vulnerable to Type I error. Thus, these results should be interpreted with caution and require replication.

Finally, generalizability of the study sample to the Australian population is limited. Majority of participants were Caucasian and highly educated. While we did capture a broader range in terms of diet quality, if we want practical implications for society, greater efforts must be made across different socioeconomic and ethnic backgrounds.

This study is strengthened by the utilization of a unique recruitment strategy and quotas to target individuals with both “optimal” and “sub-optimal” diets. This challenged the methodological flaw of previous studies [52,53] to increase the diversity of the sample with a broad range of diet quality (35 to 93 on DST). Many studies have reported a bias in the samples they have recruited, as being volunteers, they are interested in nutrition and health topics. Through this novel recruitment technique, we have been able to examine a sample who is more representative of the general population and therefore, more generalizable. Moreover, the DST provided an overall picture of diet quality and included questions regarding discretionary foods which are often overlooked in traditional diet measures [54]. Diet quality was verified by more detailed dietary analysis and objective biochemical markers. Finally, the present study considered a middle-age group which is at-risk and may be the most sensitive period for when subclinical effects associated with diet quality begin to be detected. We used age-sensitive measures and controlled for extraneous variables associated with differences in diet quality.

Previous dietary interventions have found small effect sizes, suggestive that diet alone cannot reasonably reverse age-related pathology nor act a stand-alone “cure” for mental illness. Rather, it is likely that the impact of diet on the brain may be cumulative across the lifespan. The evidence presented in this study suggests that even in mid-life, diet quality is associated with cognitive and mood outcomes. As baseline cognitive health and mood are the strongest predictors of later cognitive abilities and future psychological health, research should focus on these pre-clinical individuals to understand the relationship between diet and the brain prior to the onset of any disease. The improvement of diet quality provides great opportunity to optimize cognitive and mental health in mid-life which will be critical to significantly reducing the burden of age-associated brain disorders [40].

## 5. Conclusions

The present study examined the relationship between diet quality and psychological health in middle-aged Australian adults. As well as being a viable tool to identify nutritional risk in an Australian setting, the present study demonstrated the further application of this 20-item DST to psychological outcomes. Namely, the DST may identify those with poorer inhibitory control, greater mood disturbances and perceived stress. This tool is less costly, time consuming and burdensome than other dietary approaches typically used in epidemiology research [55] and with further validation could provide an alternative to invasive blood tests. The specific threshold for “sub-optimal” and “optimal” must be validated in an Australian population.

Analysis of diet quality as both a categorical variable with pre-defined cut-offs and a continuous variable revealed that diet may be differentially related to cognitive outcomes as opposed to mood. Future studies should consider the possibility of non-linear relationships between diet quality and psychological outcomes when analyzing and interpreting their findings. Intervention trials and longitudinal studies with long term follow ups are required to confirm these relationships between diet quality, mood, and cognition as they may follow distinct trajectories at different life stages.

## Figures and Tables

**Table 1:** Characterization of sample for all participants and by diet quality group.

	All (*n* = 141)		Optimal (*n* = 74)		Sub-Optimal (*n* = 67)		
	Mean	SD	Mean	SD	Mean	SD	*p* ^1^
Age (years)	52.84	6.87	54.09	7.14	51.47	6.34	0.024 *
Education (years)	16.94	3.36	17.09	3.18	16.78	3.57	0.576
Body Mass Index	27.26	5.22	26.28	5.19	28.36	5.06	0.018 *
MMSE	29.32	0.95	29.30	0.98	29.34	0.93	0.775
BDI-II	4.00	4.37	3.64	4.22	4.40	4.52	0.299
Waist to Hip Ratio	0.89	0.09	0.88	0.09	0.90	0.088	0.597
Diet Screening Tool	62.10	12.99	72.34	7.72	50.79	6.71	0.000 *
Gender (*n*, %)							0.929
Female	71	50.4	37	50.0	34	50.7	
Male	70	49.6	37	50.0	33	49.3	
Ethnicity (*n*, %)							0.678
Caucasian	108	76.6	55	74.3	53	79.1	
Asian	10	7.1	5	6.8	5	7.5	
Other	23	16.3	14	18.9	9	13.4	
Employment (*n*, %)							0.431
Full time	57	40.4	30	40.5	27	40.3	
Part time/Casual	50	35.5	25	33.8	25	37.3	
Studying	2	1.4	0	0.0	2	3.0	
Retired	18	12.8	12	16.2	6	9.0	
Unemployed	14	9.9	7	9.5	7	10.4	
Family history							
Cognitive disorder (% yes)	50	35.5	25	33.8	25	37.3	0.662
Psychological disorder (% yes)	23	16.3	14	18.9	9	13.4	0.379
Physical activity level (*n*, %)							0.338
Sedentary	1	0.7	0	0	1	1.5	
Insufficient	17	12.1	7	9.5	10	14.9	
Sufficient	123	87.2	67	90.5	56	83.6	
Hormonal Status (*n*, %)							0.024 *
Menstruating	25	35.2	8	21.6	17	50.0	
Peri-menopausal	8	11.3	4	10.8	4	11.8	
Post-menopausal	27	38.0	20	54.1	7	20.6	
Other ^2^	11	15.5	5	13.5	6	17.6	

^1^ Comparison by diet group was performed using ANOVA for continuous variables, and chi-square tests for categorical variables ^2^ Other included post-hysterectomy or tubes tied. SD; standard deviation. MMSE, Mini Mental State Examination; BDI-II, Beck Depression Inventory. * p < 0.05.

**Table 2:** Energy-adjusted nutrient intake derived from ASA24 recalls for all participants and by diet quality group.

		All (*n* = 140)		Optimal (*n* = 73)		Sub-Optimal (*n* = 67)		
	Units	Mean	SD	Mean	SD	Mean	SD	*p* ^1^
Kilojoules	kj	8992.39	2662.40	9000.53	2468.28	8983.53	2877.87	0.699
Kilocalories	kcal	2149.23	636.33	2151.18	589.93	2147.11	687.83	0.699
Macronutrients								
Protein	g/1000 kcal	44.87	11.89	45.10	12.00	44.62	11.86	0.325
Fat	g/1000 kcal	41.90	9.53	42.88	9.75	40.82	9.24	0.143
Carbohydrates	g/1000 kcal	97.96	21.48	94.30	21.45	101.89	20.95	0.027 *
Water	g/1000 kcal	1177.95	519.97	1241.64	603.31	1108.56	403.69	0.239
Sugar	g/1000 kcal	40.49	15.15	39.00	14.84	42.12	15.43	0.065
Fiber	g/1000 kcal	13.09	4.95	13.96	4.65	12.15	5.13	0.015 *
Vitamins								
Vitamin A	RAE/1000 kcal	510.83	334.72	532.18	352.57	487.89	315.44	0.302
Vitamin B1	mg/1000 kcal	0.91	2.04	0.71	0.33	1.13	2.93	0.758
Vitamin B2	mg/1000 kcal	1.03	0.90	0.95	0.36	1.12	1.25	0.969
Niacin	mg/1000 kcal	12.80	5.81	12.36	4.51	13.28	6.96	0.591
Vitamin B6	mg/1000 kcal	0.99	0.99	0.89	0.50	1.10	1.33	0.574
Vitamin B12	mcg/1000 kcal	2.67	4.54	1.89	0.86	3.51	6.42	0.104
Vitamin C	mg/1000 kcal	64.56	57.01	64.21	52.16	64.95	62.26	0.725
Vitamin E	mg/1000 kcal	5.91	2.04	6.34	2.16	5.43	1.80	0.007 *
Folate	mcg/1000 kcal	236.10	72.56	226.88	64.04	246.15	80.11	0.203
Minerals								
Calcium	mg/1000 kcal	407.94	156.33	414.47	163.17	400.83	149.43	0.955
Iron	mg/1000 kcal	5.89	2.06	6.05	1.88	5.71	2.24	0.088
Magnesium	mg/1000 kcal	198.63	63.21	215.72	64.03	180.01	57.16	0.001 *
Phosphorus	mg/1000 kcal	728.32	140.04	744.26	139.24	710.94	139.87	0.094
Potassium	mg/1000 kcal	1537.99	370.29	1591.44	355.62	1479.75	379.74	0.119
Sodium	mg/1000 kcal	1223.81	487.70	1174.29	541.52	1277.76	418.76	0.315
Zinc	mg/1000 kcal	5.23	1.64	5.46	1.79	4.99	1.45	0.043 *
Selenium	mcg/1000 kcal	46.52	20.34	48.84	23.78	44.01	15.64	0.457
Fats								
Omega 3 (*n*-3)	% of energy	0.08	0.03	0.08	0.04	0.07	0.03	0.122
Omega 6 (*n*-6)	% of energy	0.60	0.26	0.67	0.29	0.53	0.21	0.001 *
*n*-6: *n*-3	ratio	8.31	3.34	8.99	3.68	7.56	2.75	0.044 *
PUFA	% of energy	0.72	0.29	0.79	0.52	0.64	0.25	0.001 *
MUFA	% of energy	1.65	0.49	1.73	0.52	1.57	0.45	0.017 *
SFA	% of energy	1.47	0.47	1.41	0.46	1.53	0.47	0.082
PUFA: SFA	ratio	0.45	0.16	0.47	0.18	0.41	0.13	0.085

^1^ ANCOVA models controlled for age and BMI. SD, standard deviation; PUFA, polyunsaturated fatty acid; MUFA, monounsaturated fatty acid; SFA, saturated fatty acid. * p < 0.05.

**Table 3:** Biochemical markers for all participants and by diet quality group.

	All			Optimal			Sub-Optimal			
	*n*	Mean	SD	*n*	Mean	SD	*n*	Mean	SD	*p* ^1^
Saturated fats (%)	118	43.15	0.65	65	43.03	0.67	53	43.31	0.59	0.012 *
Trans-saturated fats (%)	118	0.42	0.14	65	0.42	0.15	53	0.43	0.12	0.670
Omega 3 (%)	118	8.72	1.31	65	8.58	1.31	53	8.89	1.31	0.089
Omega 3 Index ^2^	118	5.63	1.26	65	5.54	1.26	53	5.73	1.26	0.191
EPA (%)	118	0.99	0.34	65	0.98	0.36	53	1.00	0.32	0.480
DHA (%)	118	4.64	1.07	65	4.57	1.05	53	4.73	1.10	0.198
Omega 6 (%)	118	30.39	1.58	65	30.57	1.61	53	30.17	1.54	0.086
*n*-6: *n*-3 ratio	118	3.59	0.70	65	3.67	0.77	53	3.48	0.60	0.052
Vitamin B12 (pmol/L)	139	321.09	114.00	74	307.91	109.00	65	336.09	119.00	0.153
Vitamin B6 (nmol/L)	136	102.32	62.00	70	111.29	72.00	66	92.80	49.00	0.030 *
Homocysteine (µmol/L)	139	10.28	2.84	74	10.02	2.66	65	10.57	3.02	0.056
RBC folate (nmol/L)	62	1242.50	260.11	43	1287.51	244.24	19	1140.63	274.56	0.026 *

^1^ ANCOVA models controlled for age and BMI. ^2^ Omega 3 Index is total value of eicosapentaenoic acid and docosahexaenoic acid. % values for fatty acids indicate percentage of total fatty acids. SD; standard deviation. * p < 0.05.

**Table 4:** Cognitive and mood outcomes for all participants and by diet quality group.

	All			Optimal			Sub-Optimal			
	*n*	Mean	SE	*n*	Mean	SE	*n*	Mean	SE	*p* ^1^
Stroop Processing	139	0.05	0.07	72	0.19	0.10	67	−0.10	0.09	0.013 *
Reaction and Decision Speed	140	0.02	0.07	74	0.06	0.11	66	−0.02	0.10	0.337
Visual Processing	140	0.00	0.07	73	0.02	0.11	67	−0.02	0.10	0.586
Spatial Working Memory	141	0.00	0.08	74	−0.11	0.12	67	0.12	0.12	0.342
Perceived Stress ^2^	141	11.00	(7.00–15.00)	74	10.00	(6.00–15.00)	67	12.00	(8.00–16.00)	0.527
Total Mood Disturbance ^2^	141	−1.00	(−9.00–14.00)	74	−3.00	(−11.00–9.00)	67	2.00	(−4.00–20.00)	0.177

^1^ ANCOVA models controlled for age, gender, years of education and BMI. ^2^ median and interquartile range presented. SE; standard error. * p < 0.05. Z scores are presented for each of the cognitive domains with higher scores indicative of better performance. “Stroop Processing” is the difference between Incongruent and Congruent Stroop performance; “Reaction & Decision Speed” is derived from the average performance for Simple Reaction Time and Choice Reaction Time tasks; “Visual Processing” is derived from the average performance for Immediate Recognition, Delayed Recognition and Contextual Memory tasks, and “Spatial Working Memory” is derived from the average performance for Spatial Working Memory task. For mood outcomes (Perceived Stress, Total Mood Disturbance), lower scores indicate better mood.

**Table 5:** Standardized weights of the relationship between diet quality (DST) and cognitive and mood outcomes after adjusting for covariates.

	DST Score			
	*n*	β	F Change	*p* ^1^
Stroop Processing	139	0.127	1.941	0.166
Reaction and Decision Speed	140	0.063	0.509	0.477
Visual Processing	140	0.089	1.035	0.311
Spatial Working Memory	141	0.051	0.356	0.511
Perceived Stress	141	−0.191	4.729	0.031 *
Total Mood Disturbance	141	−0.269	9.636	0.002 *

^1^ Regression model adjusted for: age, gender, years of education, BMI. DST; Diet Screening Tool. * p < 0.05.
